# RDW Predicts Fibrosis in Patients with Chronic Hepatitis B Having Persistently Normal ALT Levels

**DOI:** 10.4314/ejhs.v33i4.5

**Published:** 2023-07

**Authors:** Basak Yilmaz Guller, Erdinc Gulumsek, Hilmi Erdem Sumbul, Begum Seyda Avci, Adnan Tas

**Affiliations:** 1 Department of Internal Medicine, University of Health Sciences - Adana City Research and Training Hospital, Adana, Turkey; 2 Department of Gastroenterology, University of Health Sciences - Adana City Research and Training Hospital, Adana, Turkey

**Keywords:** Chronic Hepatitis B, Liver fibrosis, APRI, FIB-4, RDW, MPV

## Abstract

**Background:**

There are studies on the determination of hepatic fibrosis with noninvasive markers but data about liver biopsy results and noninvasive markers in patients with chronic hepatitis B (CHB) are limited. The aim of this study is to determine the relationship between pathological findings and noninvasive markers, and to determine the marker that predicts fibrosis in patients with consistently normal serum alanine aminotransferase (ALT) levels, diagnosed with CHB and undergoing liver biopsy.

**Methods:**

A total of 122 patients with CHB, 29 of them with HbeAg (+), aged 30 years and older, HBV DNA > 2000 IU / ml, and serum ALT levels measured four times in the last year, were consistently normal, and 93 of them with HbeAg (-) were included in the study. Demographic characteristics of patients, laboratory parameters, histological activity index (HAI) and fibrosis values obtained in liver biopsy, and noninvasive markers (AP (age-platelet) index, APRI (AST/Platelet ratio) and FIB-4 score, neutrophil/lymphocyte ratio, mean platelet volume (MPV) and erythrocyte distribution width (RDW) were recorded.

**Results:**

The relationship between RDW value and fibrosis was statistically significant in the HbeAg (+) group (p<0.001). The relationship between AP index, APRI and FIB-4 score, neutrophil/lymphocyte ratio and MPV with fibrosis was not statistically significant (>0.05 for each).

**Conclusion:**

It has been shown that the RDW value can be used to predict fibrosis in CHB patients with normal ALT and HbeAg (+), and the cut-off value for RDW is 12.

## Introduction

Chronic hepatitis B (CHB) is an infection that can have consequences such as liver cirrhosis, hepatocellular carcinoma and death. Despite the current medical facilities, CHB treatment does not have a clear starting points and end points (1,2).

The treatment and follow-up criteria of patients diagnosed with CHB are determined by the national and international guidelines.

According to these guidelines, CHB patients who have HBV DNA levels above 2000 IU/ml should be evaluated with particular markers to assess their suitability for treatment. In patients with consistently normal serum alanine aminotransferase (ALT) levels but HBV DNA levels above 2000 IU/ml, performing liver biopsy is indicated if the patient is over 30 years old or if the patient is under 30 years old and has a suspected liver injury (prolonged prothrombin time, low albumin, thrombocytopenia). After pathological assessment of the liver biopsy, if the Histological Activity Index (HAI) is ≥6 or the Fibrosis is ≥2 according to the ISHAK scoring system, the treatment is initiated (3). Histopathological examination of the liver biopsy specimen is currently the gold standard for staging hepatic fibrosis. Although great progress has been made regarding non invasive markers, current studies do not yet directly supported by the histological analysis (4). In addition; despite the relationship between liver biopsy results and non-invasive markers in patients with chronic liver disease is well known, the data on CHB regarding this subject is limited.

In this study; patients who wer 30 years old and over and had HBV DNA > 2000 IU / ml, whose serum ALT levels consistently normal and diagnosed with CHB by liver biopsy were examined. Our aim was to investigate the relationship between HAI and fibrosis scores obtained in liver biopsy and noninvasive markers (AP (age-platelet) index, APRI (Aspartate aminotransferase (AST)-platelet ratio) score, FIB-4 score, neutrophil/lymphocyte ratio (NLR), mean platelet volume (MPV), and red cell distribution width (RDW) in order to detect CHB-related damage in the liver and to identify markers that predict fibrosis.

## Material and Method

**Study design and data collection**: Our study was conducted by scanning a total of 1225 patients who applied to the gastroenterology clinic of Health Science University, Adana Numune Training and Research Hospital between 01.01.2010 - 31.12.2018 and underwent liver biopsy. A total of 122 patients, 29 of whom were HbeAg positive and 93 were Anti-Hbe positive, who met the inclusion criteria were included in the study. Among the patients diagnosed with CHB, the patients who were suitable according to the criteria described below were included in the study.

Inclusion criteria for the study were; a) patients who were diagnosed with CHB, were HbeAg positive or Anti-Hbe positive and who did not receive any treatment due to CHB, b) patients with ALT levels within normal limits in all measurements taken within the last year (Normal ALT level was determined as 40 U/L), c) patients with HBV DNA level >2000 IU/ml, d) patients whose liver biopsy was performed and HAI and fibrosis scores were determined according to the Modified Knodell and Ishak scoring system, e) patients whose ALT, AST, MPV, RDW, neutrophil, lymphocyte and platelet values were registered on the system at the time of biopsy in order to calculate the non-invasive markers we have determined. Exclusion criteria for the study were; a) patients with an additional disease that may cause chronic liver disease, b) patients who were clinically and biochemically diagnosed with cirrhosis, c) patients with coinfection such as HCV, HIV, HDV, d) patients who have received treatment for CHB before, e) Patients with iron deficiency and megaloblastic anemia.

**Calculation of scores**: The APRI (AST/Platelet ratio) score of the patients were calculated using the ((AST/AST upper limit value of normal) / Plt 10^9/L) *100 formula and the FIB-4 score was calculated using the (age*AST) / (Platelet*√ALT) formula. AP (age-platelet) index is calculated as the sum of age and platelet points who are determined as follows: a) for age; 0 points for those 30 and under, 1 point for those between 31 and 40, 2 points for those between 41 and 50, 3 points for those between 51 and 60, 4 points for those between 61 and 70, and 5 points for those 71 and over b) for platelet; 0 points for those with a platelet count above 225 thousand, 1 point for those between 200-224 thousand, 2 points for those between 175-199 thousand, 3 points for those between 150-174 thousand, 4 points for those between 125-149 thousand and 5 points for those 124 thousand and below

**Statistical evaluation**: All analyzes were performed using the SPSS 22.0 (Chicago, IL, USA) statistical software package. The variables were divided into two groups as categorical and continuous. The distribution of continuous variables was evaluated with the Kolmogorov-Smirnov test. Continuous variables were expressed as mean ± SD. Student's t-test was used to compare HbeAg (+) and Anti-Hbe (+) groups with low and high fibrosis rates. ROC analysis was used to determine the relationship between fibrosis and the NLR, MPV, RDW, AP Index, APRI and FIB-4 scores of the patients included in the study. Using the data obtained in the ROC analysis, cut-off, specificity, sensitivity, positive predictive and negative predictive values were determined for patients with fibrosis score above 2, separately in patients with HbeAg (+) and Anti-Hbe (+). A p value of <0.05 was considered statistically significant in all analyzes.

**Ethical approval**: The ethics committee of the Adana City Research and Training Hospital Hospital, Ethics Committee approved the study. This manuscript was carried out in accordance with the Declaration of Helsinki and Good Clinical Practice guidelines.

## Results

The demographic, hematological and biochemical data of the HbeAg (+) and Anti-Hbe (+) patients in the study, and the statistical evaluation of the patients' NLR and AP indices and HAI, fibrosis, APRI and FIB-4 scores between the groups are summarized in Table 1. As shown in Table 1; there were statistically significant differences in terms of ALT, AST, lymphocyte counts and APRI scores between the groups, but there were no statistically significant differences in terms of other parameters.

**Table 1:** Examination of demographic, laboratory and non-invasive values of all patients and examination of patients in terms of parameters associated with fibrosis

	HbeAg (+) all patients	Anti-Hbe (+) all patients	P value
Variable	(n:29)	(n:93)	
Sex (female)	14 (48.3%)	40 (43.0%)	0.387
Age (year)	42.0±10.66	45.66±10.95	0.117
ALT (u/L)	29.50±7.59	23.21±8.19	**<0.001**
AST (u/L)	27.04±6.45	22.15±6.39	**<0.001**
Platelets (10^3/μl)	244.0±71.69	242.95±62.49	0.939
Lymphocyte (10^3/μl)	2.67±0.82	2.22±0.59	**0.002**
Neutrophil (10^3/μl)	4.45±1.19	4.08±1.23	0.155
MPV (fL)	9.37±1.71	9.31±1.53	0.858
RDW (%)	14.39±1.37	14.09±1.68	0.385
APRI	0.30±0.12	0.24±0.11	**0.017**
FIB-4	0.96±0.48	0.96±0.53	0.991
NLR	1.79±0.63	1.95±0.80	0.309
AP index	2.48±1.70	2.80±1.83	0.416
Fibrosis score	2.00±1.03	2.03±1.16	0.894
HAI	6.34±2.24	5.94±2.62	0.462

	Fibrosis <2 all patients	Fibrosis ≥2 all patients	P value

Variable	(n: 36)	(n: 86)	
Age (year)	42.11±9.73	45.91±11.28	0.081
ALT (u/L)	22.30±8.12	25.71±8.45	**0.042**
AST (u/L)	21.52±6.34	24.06±6.76	0.057
Platelets (10^3/μl)	239.28±52.45	244.84±69.13	0.666
Lymphocyte (10^3/μl)	2.23±0.67	2.37±0.68	0.292
Neutrophil (10^3/μl)	4.34±1.30	4.09±1.20	0.313
MPV (fL)	9.57±1.30	9.22±1.66	0.264
RDW (%)	13.69±1.35	14.36±1.68	**0.036**
APRI	0.24±0.11	0.26±0.11	0.250
FIB-4	0.88±0.42	1.00±0.55	0.259
NLR	2.07±0.72	1.85±0.78	0.165
AP index	2.47±1.55	2.83±1.89	0.325
HAI	4.41±1.36	6.72±2.60	**<0.001**

ALT: Alanine aminotransferase, AST: Aspartate aminotransferase, MPV: Mean platelet volume, RDW: Erythrocyte distribution width, APRI: AST-to-Platelet ratio index. FIB-4: Fibrosis-4, NLR: Neutrophil to lymphocyte ratio, AP index: Age- Platelet index, HAI: histological activity index

All patients included in our study were divided into two groups as fibrosis score <2 and fibrosis score ≥2. 86 patients with fibrosis score ≥2 and 36 patients with fibrosis score <2 were compared in terms of age, ALT, AST, MPV, RDW, platelet, lymphocyte and neutrophil counts, APRI scores and FIB4 scores, NLR and AP indices and HAI. The statistical evaluation between the groups is summarized in Table 1. As shown in Table 1; there were statistically significant differences in terms of ALT, RDW and HAI scores between the groups, but there were no statistically significant differences in terms of other parameters.

**Figure 1:**
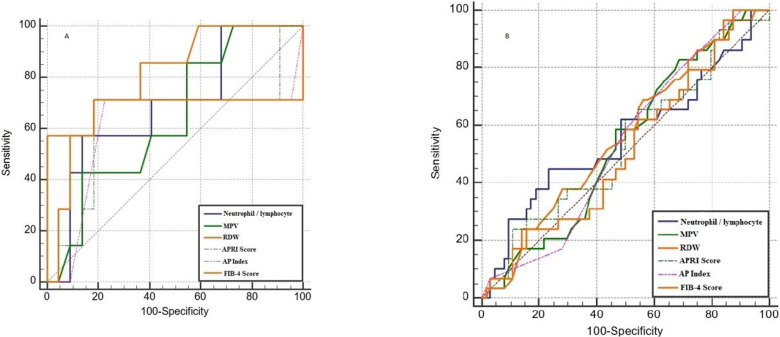
ROC curve of HbeAg (+) patients with fibrosis ≥2 (A) and ROC curve of Hbeag (-) AntiHbe (+) patients with fibrosis ≥2 (B)

The parameters that may be related to the fibrosis levels of the HbeAg (+) and AntiHbe (-) patient group and the HbeAg (-) and AntiHbe (+) patient group were compared. In the HbeAg (+) group, patients with fibrosis score ≥2 (22 patients) and patients with fibrosis score <2 (7 patients) were analyzed and compared in terms of age, ALT, AST, PLT, MPV, RDW, lymphocyte and neutrophil counts, APRI and FIB4 scores, NLR and AP indexes and HAI scores. The statistical evaluation between the groups is summarized in Table 2. As shown in Table 2; there were statistically significant differences in terms of neutrophil count, RDW and HAI scores between the groups, but there were no statistically significant differences in other parameters. ROC analysis of parameters that can be used to identify the patients with fibrosis scores ≥2 in the HbeAg (+) group are summarized in Table 3 and Figure 1 (A). In the ROC analysis performed to identify patients with fibrosis scores of 2 or higher in the HbeAg (+) group; AUC value of RDW was 0.841, the cut-off was 12, the specificity was 100.0 (84.6-100.0), the sensitivity was 57.14 (18.4- 90.1) and PPV/ NPV rate was 100.0 / 88.0.

**Table 2:** Demographic, non-invasive and laboratory findings in patients with HbeAg (+) fibrozis <2 and ≥2

Variable	<2 fibrozis (n: 7)	≥2 fibrozis (n: 22)	p
Age (year)	40.57±13.50	42.45±9.93	0.692
ALT (u/L)	29.14±6.74	29.61±7.99	0.888
AST (u/L)	24.28±4.75	27.92±6.76	0.199
Platelets (10^3/μl)	255.57±83.04	240.32±69.45	0.633
Lymphocyte (10^3/μl)	2.70±0.67	2.66±0.88	0.907
Neutrophil (10^3/μl)	5.55±1.43	4.10±0.88	**0.003**
MPV (fL)	10.0±1.42	9.17±1.77	0.273
RDW (%)	13.21±1.20	14.76±1.21	**0.007**
APRI	0.26±0.12	0.31±0.12	0.368
FIB-4	0.89±0.69	0.99±0.41	0.662
NLR	2.09±0.48	1.69±0.65	0.144
AP index	2.43±2.44	2.50±1.47	0.925
HAI	4.42±0.53	6.95±2.23	**0.007**

ALT: Alanine aminotransferase, AST: Aspartate aminotransferase, MPV: Mean platelet volume, RDW: Erythrocyte distribution width, APRI: AST-to-Platelet ratio index. FIB-4: Fibrosis-4, NLR: Neutrophil to lymphocyte ratio, AP index: Age- Platelet index, HAI: histological activity index

**Table 3:** ROC analysis for the detection of HbeAg (+) patients with fibrozis ≥2

Variable	AUC	Cutoff	Spesitive (95%-Cl %)	Sensitive (95%-Cl %)	P
MPV	0.640	>8.7	45.45 (24.4-67.8)	85.71 (42.1-99.6)	0.234
RDW	0.841	≤12.6	100.0 (84.6-100.0)	57.14 (18.4-90.1)	**0.001**
APRI	0.636	≤0.23	81.82 (59.7-94.8)	71.43 (29.0-96.3)	0.370
AP index	0.607	≤1	77.27 (54.6-92.2)	71.43 (29.0-96.3)	0.501
FIB-4	0.649	≤0.64	81.82 (59.7-94.8)	71.43 (29.0-96.3)	0.387
NLR	0.688	>2	86.36 (65.1-97.1)	57.1 (18.4-90.1)	0.122

MPV: Mean platelet volume, RDW: Erythrocyte distribution width, APRI: AST-to-Platelet ratio index. FIB-4: Fibrosis-4, NLR: Neutrophil to lymphocyte ratio, AP index: Age- Platelet index

In the HbeAg (-) and AntiHbe (+) group, 64 patients with fibrosis scores ≥ 2 and 29 patients with fibrosis scores <2 were included in the study. These patients were analyzed and compared in terms of age, ALT, AST, PLT, MPV, RDW, lymphocyte and neutrophil counts, APRI and FIB4 scores, NLR and AP indexes and HAI scores. There were statistically significant differences in terms of ALT and HAI scores between the groups, but there were no statistically significant differences in other parameters (Table 4). ROC analysis of the parameters of patients with fibrosis ≥ 2 in HbeAg (-) and AntiHbe (+) group are summarized in Table 5 and Figure 1 (B).

**Table 4:** Demographic, non-invasive and laboratory findings in patients with HbeAg (-) AntiHbe (+) fibrozis <2 and ≥2

Variable	<2 fibrozis (n: 29)	≥2 fibrozis (n: 64)	^p^
Age (year)	42.48±8.87	47.09±11.54	0.060
ALT (u/L)	20.65±7.63	24.37±8.23	0.042
AST (u/L)	20.86±6.56	22.73±6.28	0.193
Platelets (10^3/μl)	235.34±43.34	246.39±69.50	0.433
Lymphocyte (10^3/μl)	2.11±0.62	2.27±0.58	0.236
Neutrophil (10^3/μl)	4.05±1.10	4.09±1.30	0.884
MPV (fL)	9.46±1.28	9.24±1.63	0.508
RDW (%)	13.80±1.38	14.22±1.80	0.274
APRI	0.23±0.10	0.25±0.11	0.571
FIB-4	0.88±0.35	1.00±0.59	0.303
NLR	2.06±0.78	1.91±0.82	0.410
AP index	2.48±1.32	2.94±2.01	0.270
HAI	4.41±1.50	6.64±2.73	<0.001

ALT: Alanine aminotransferase, AST: Aspartate aminotransferase, MPV: Mean platelet volume, RDW: Erythrocyte distribution width, APRI: AST-to-Platelet ratio index. FIB-4: Fibrosis-4, NLR: Neutrophil to lymphocyte ratio, AP index: Age- Platelet index, HAI: histological activity index

**Table 5:** ROC analysis for the detection of HbeAg (-) Anti-Hbe (+) patients with fibrozis ≥2

Variable	AUC	Cutoff	Spesitive (95%-Cl %)	Sensitive (95%-Cl %)	p
MPV	0.535	>8.2	31.25 (20.2-44.1)	82.76 (64.2-94.2)	0.571
RDW	0.558	≤14	43.75 (31.4-56.7)	68.97 (49.2-84.7)	0.358
APRI	0.536	≤0.15	89.06 (78.8-95.5)	24.14 (10.3-43.5)	0.581
AP index	0.533	≤1	71.87 (59.2-82.4)	17.24 (5.8-35.8)	0.580
FIB-4	0.514	≤1.71	12.5 (5.6-23.2)	100.0 (88.1-100.0)	0.827
NLR	0.557	>2.15	76.56 (64.3-86.2)	44.83 (26.4-64.3)	0.411

MPV: Mean platelet volume, RDW: Erythrocyte distribution width, APRI: AST-to-Platelet ratio index. FIB-4: Fibrosis-4, NLR: Neutrophil to lymphocyte ratio, AP index: Age- Platelet index

## Discussion

In our study, when HbeAg (+) Anti-Hbe (-) patients with consistently normal ALT levels and patients with HbeAg (-) Anti-Hbe (+) were evaluated conjointly, the relationship between fibrosis and RDW, which is one of the parameters that can be a noninvasive fibrosis marker, was found to be statistically significant. However, when both patient groups were considered separately, the relationship between RDW and fibrosis was found to be statistically significant also in the HbeAg (+) patient group. This relationship could not be demonstrated in the Anti-Hbe (+) patient group. In this study, the relationship between fibrosis and MPV, NLR, AP index, APRI and FIB4 score parameters, which were predicted to be used as fibrosis markers, was not found statistically significant.

RDW is an objective indicator of anisocytosis. In recent years, a number of reports have been published suggesting that RDW is a prognostic marker in various disorders (5).

In a study by Mengjie Zhu et al., it was determined that the RDW value in the HbeAg (+) patient group was statistically higher than the HbeAg (-) patients and healthy adults. Again in the same study, when HBV-associated cirrhosis patients were selected as the patient group and CHB and inactive carriers were selected as the control group, the AUC value for RDW was 0.66, the cut-off value was 13.05, the sensitivity 66.91% and the specificity 64.69% (6).

Huang et al. found that RDW value was significantly higher in hepatitis B-associated cirrhosis patients than CHB and healthy control groups, and there was a significant correlation between RDW value and Child Pugh classification and MELD scores. They also suggested that RDW is a new marker for assessing the severity of HBV-related liver diseases (7).

In the meta-analysis study of the relationship between hepatitis B-related liver diseases and RDW conducted by Fan X. et al., CHB patients and healthy controls were compared, and RDW values were found to be significantly higher than healthy controls, and, it also found to be significantly higher in patients with acute-on-chronic liver failure and liver cirrhosis patients (8).

In our study, a significant correlation was found between fibrosis levels and RDW, independent of HbeAg (+) and HbeAg status of patients with chronic hepatitis B with normal ALT levels. When our study was evaluated in terms of RDW, we obtained similar results to previous studies. A number of studies have shown that RDW may be associated with disease activity and is an indicator of inflammation.

It is thought that there is a chronic inflammation in chronic hepatitis B and this inflammation causes an increase in RDW levels by affecting erythrocyte lifespan as a result of its effects on iron metabolism and bone marrow (9). However, the absence of a significant relationship between RDW and patients with Anti-Hbe (+) and fibrosis score of 2 and above cannot be explained by this mechanism. We think that more comprehensive analyzes are needed in this regard.

In the study cunducted by Xiao G. et al., when the sensitivity and specificity of APRI in predicting fibrosis in CHB were evaluated, when the cut-off value was taken as 0.5, APRI had a sensitivity of 70.0% and a specificity of 60.0%, and when the cut-off value was taken as 1.5, sensitivity and specificity were shown to be 34.1% and 89.5%, respectively (10). In a study conducted by Huang D et al., it was shown that the APRI score was statistically significantly higher in the fibrosis 2 and above group (11). In the study conducted by Tan YW et al. with patients diagnosed with CHB, no difference was found in the group with consistently normal ALT, but the relationship between APRI and fibrosis was statistically significant in the two patient groups with intermittent ALT levels less than twice ULN or more, and in this group the AUC value was calculated as 0.735, the specificity as 83.7%, the sensitivity as 85.7% and the cut off value as 1.26 (12).

In our study, the APRI score was found to be statistically higher in the HbeAg (+) group than in the HbeAg (-) group. However, when the relationship between fibrosis and APRI was examined, no statistically significant relationship was found in the comparison made in both HbeAg (+) and HbeAg (-) patient groups separately and in all patients independent of HbeAg status. All these data show that APRI does not have sufficient sensitivity and specificity to predict fibrosis in patients with consistently normal ALT levels.

X.Z. Yang et al's study on FIB-4 score, another parameter studied as an indicator of noninvasive fibrosis, was found to be significantly higher in cases with fibrosis stage 2 and above (13). Taneja S et al. pointed out that the sensitivity of FIB-4 value for the diagnosis of cirrhosis was 57.9%, specificity was 95.7%, and AUC value was 0.90 in their study to predict the treatment response and fibrosis level of patients with chronic hepatitis C using noninvasive methods. They also reported that the sensitivity and specificity of FIB-4 for significant fibrosis were 73.6% and 68.3%, respectively, with an AUC of 0.79 (14). In our study, no statistically significant results were obtained when fibrosis and FIB-4 values were compared in two patient groups - HbeAg (+) and HbeAg (-) - and in all patients independent of HbeAg status. This may be explained by the fact that the patient group with normal ALT levels was examined and the mean fibrosis scores were low in our study. However, more studies are needed to use the FIB-4 score as a fibrosis marker in CHB.

In the study conducted by Poynard and Bedossa, it was shown that the AP index are independent variables that correlate with fibrosis and histological activity index (15) and, In the study of Chrostek L. et al., it was determined that the AP index is a weak marker to show fibrosis (16). In our study, however, AP index was not found to be correlated with fibrosis in all patient groups. This may be explained by the fact that the AP index is a weak indicator of fibrosis or the lower mean age of the patients in our study.

MPV, which defines platelet size, is not only a marker of platelet function and activity, but is also accepted as a new index of inflammation (17). Ceylan et al. stated that MPV is an independent variable that indicates the severity of inflammation rather than indicating liver fibrosis in patients with CHB (18). In our study, the relationship between MPV and liver fibrosis was not found to be significant. When the two patient groups - HbeAg (+) and HbeAg (-) - were examined separately and conjointly, we concluded that MPV could not be used as a parameter that can show liver damage.

Although NLR is a prognostic factor in various diseases, data in the literature are contradictory (19). In the study by Kekili et al., patients diagnosed with CHB were examined in two groups according to their fibrosis levels as fibrosis <2 and fibrosis ≥2, and it was found that NLR showed a negative correlation with fibrosis level in patients with CHB (20). Celikbilek M. et al. evaluated 89 patients with CHB diagnosed with liver biopsy and 43 healthy control groups and showed that there was no statistically significant difference in NLR values between the two groups (21). In our study, no significant relationship was found between NLR and fibrosis.

We found that RDW value can be used as a noninvasive fibrosis marker in the estimation of fibrosis and the cut-off value of RDW is 12 in the HbeAg (+) CHB patient group with normal ALT. The limitations of our study were being a single-center, cross-sectional study and having limited number of patients included. Also, biopsy results being interpreted by a single individual were also a limitation for our study.
